# Xpert MTB/RIF Ultra Is Highly Sensitive for the Diagnosis of Tuberculosis Lymphadenitis in a High-HIV Setting

**DOI:** 10.1128/JCM.01316-21

**Published:** 2021-11-18

**Authors:** Stephanie Minnies, Byron W. P. Reeve, Loren Rockman, Georgina Nyawo, Charissa C. Naidoo, Natasha Kitchin, Cornelia Rautenbach, Colleen A. Wright, Andrew Whitelaw, Pawel Schubert, Robin M. Warren, Grant Theron

**Affiliations:** a DSI-NRF Centre of Excellence for Biomedical Tuberculosis Research, South African Medical Research Council Centre for Tuberculosis Research, Division of Molecular Biology and Human Genetics, Faculty of Medicine and Health Sciences, Stellenbosch University, Cape Town, Republic of South Africa; b Department of Psychiatry, Faculty of Medicine and Health Sciences, Stellenbosch University, Cape Town, Republic of South Africa; c Division of Medical Microbiology, Faculty of Medicine and Health Sciences, Stellenbosch University, Cape Town, Republic of South Africa; d National Health Laboratory Services, Cape Town, Republic of South Africa; e Division of Anatomical Pathology, Faculty of Medicine and Health Sciences, Stellenbosch University, Cape Town, Republic of South Africa; University of Manitoba

**Keywords:** Xpert MTB/RIF Ultra, diagnostic accuracy, tuberculosis lymphadenitis

## Abstract

Tuberculosis lymphadenitis (TBL) is the most common extrapulmonary tuberculosis (EPTB) manifestation. Xpert MTB/RIF Ultra (Ultra) is a World Health Organization-endorsed diagnostic test, but performance data for TBL, including on noninvasive specimens, are limited. Fine-needle aspiration biopsy specimens (FNABs) from outpatients (≥18 years) with presumptive TBL (*n* = 135) underwent (i) routine Xpert MTB/RIF testing (later with Ultra once programmatically available), (ii) MGIT 960 culture (if Xpert or Ultra negative or rifampicin resistant), and (iii) study Ultra testing. Concentrated paired urine specimens underwent Ultra testing. Primary analyses used a microbiological reference standard (MRS). In a head-to-head comparison (*n* = 92) of an FNAB study Ultra and Xpert, Ultra had increased sensitivity (91% [95% confidence interval: 79, 98] versus 72% [57, 84]; *P* = 0.016) and decreased specificity (76% [61, 87] versus 93% [82, 99]; *P* = 0.020) and diagnosed patients not on treatment. Neither HIV nor alternative reference standards affected sensitivity and specificity. In patients with both routine and study Ultra tests, the latter detected more cases (+20% [0, 42]; *P* = 0.034), and false-negative study Ultra results were more inhibited than true-positive results. Study Ultra false positives had less mycobacterial DNA than true positives (trace-positive proportions, 59% [13/22] versus 12% [5/51]; *P* < 0.001). “Trace” exclusion or recategorization removed potential benefits offered over Xpert. Urine Ultra tests had low sensitivity (18% [7, 35]). Ultra testing on FNABs is highly sensitive and detects more TBL than Xpert (Ultra still missed some cases due in part to inhibition). Patients with FNAB Ultra-positive “trace” results, most of whom will be culture negative, may require additional clinical investigation. Urine Ultra testing could reduce the number of patients needing invasive sampling.

## INTRODUCTION

Tuberculosis (TB) is a leading cause of morbidity and mortality globally. In 2019, extrapulmonary TB (EPTB) represented 16% of new TB cases reported (1) and, in HIV-positive populations, can account up to 50% of all TB (2). TB lymphadenitis (TBL) accounts for 35% of all EPTB (3, 4). South Africa, with a high TB and HIV burden (1), is particularly affected by EPTB and TBL.

TBL is typically diagnosed by examining fine-needle aspiration biopsy specimens (FNABs) from affected lymph nodes. This requires specialized sampling and facilities, and tests have suboptimal sensitivity (5). One widely used test is Xpert MTB/RIF (Xpert; Cepheid, USA), a semiautomated real-time PCR that rapidly detects Mycobacterium tuberculosis complex (MTBC) DNA and rifampicin resistance (6, 7). A systematic review and meta-analysis showed heterogeneity in the sensitivity of FNAB Xpert versus microbiological (83% [95% confidence interval: 71, 91]) and composite (81% [72, 88]) reference standards (8). Specificities were 94% (88, 97) and 99% (95, 100), respectively (8). Most EPTB diagnostic algorithms recommend culture after a negative Xpert result (9); however, this creates delay. Better TBL tests are needed.

One potential test is the Xpert MTB/RIF Ultra (Ultra), which offers improved sensitivity over Xpert for pulmonary TB, partly enabled by, in addition to *rpoB*, amplification of multicopy insertion elements (IS*6110*, IS*1081*) (10). Data on Ultra for TBL are emerging: one retrospective evaluation tested 10 Xpert-negative, culture-positive FNABs and found half to be Ultra positive (11); another retrospective evaluation (*n* = 25) reported sensitivity and specificity of 94% (95% confidence interval: 71, 77)) and 100% (63, 72), respectively (12); and a prospective evaluation (*n* = 73) reported a sensitivity and specificity of 78% (40, 97) and 78% (66, 87), respectively (13). No studies included head-to-head Xpert and Ultra data. Additionally, since Ultra’s advent, algorithms for TBL diagnosis remain essentially unchanged from the Xpert era—culture is still recommended in Ultra-negative patients. Whether this is needed or, conversely, if culture is needed to confirm positive Ultra results due to specificity concerns associated with the new trace semiquantitation category (10, 14) requires investigation.

Lastly, FNABs are rarely collected in primary care; patients are referred to district or tertiary facilities, resulting in care cascade gaps (15). If an Ultra has high sensitivity and specificity on an easily accessible fluid like urine, the need for invasive sampling could be mitigated, potentially drastically reducing provider and patient economic and time costs, including those associated with referral. To our knowledge, urine Ultra testing for TBL specifically is unevaluated.

We evaluated the head-to-head diagnostic accuracy of Xpert and Ultra on FNABs and Ultra on urine specimens in patients with presumptive TBL in a tertiary hospital in a high-HIV setting in South Africa. We hypothesized that Ultra would show improved sensitivity compared to Xpert.

## MATERIALS AND METHODS

### Ethics statement.

The study was approved by the Stellenbosch University Human Research Ethics Committee and Tygerberg General Hospital (TGH) (both N16/04/050).

### Patient recruitment.

One hundred thirty-five outpatients (≥18 years) with presumptive TBL (swollen lymph nodes) undergoing routine referral and investigation at a tertiary referral clinic at TGH in Cape Town, South Africa, were consecutively recruited from 25 January 2017 to 12 March 2019 and provided FNABs and urine. Patients who received TB treatment ≤60 days prior were excluded.

### Fine-needle aspirate collection.

**FIG 1:**
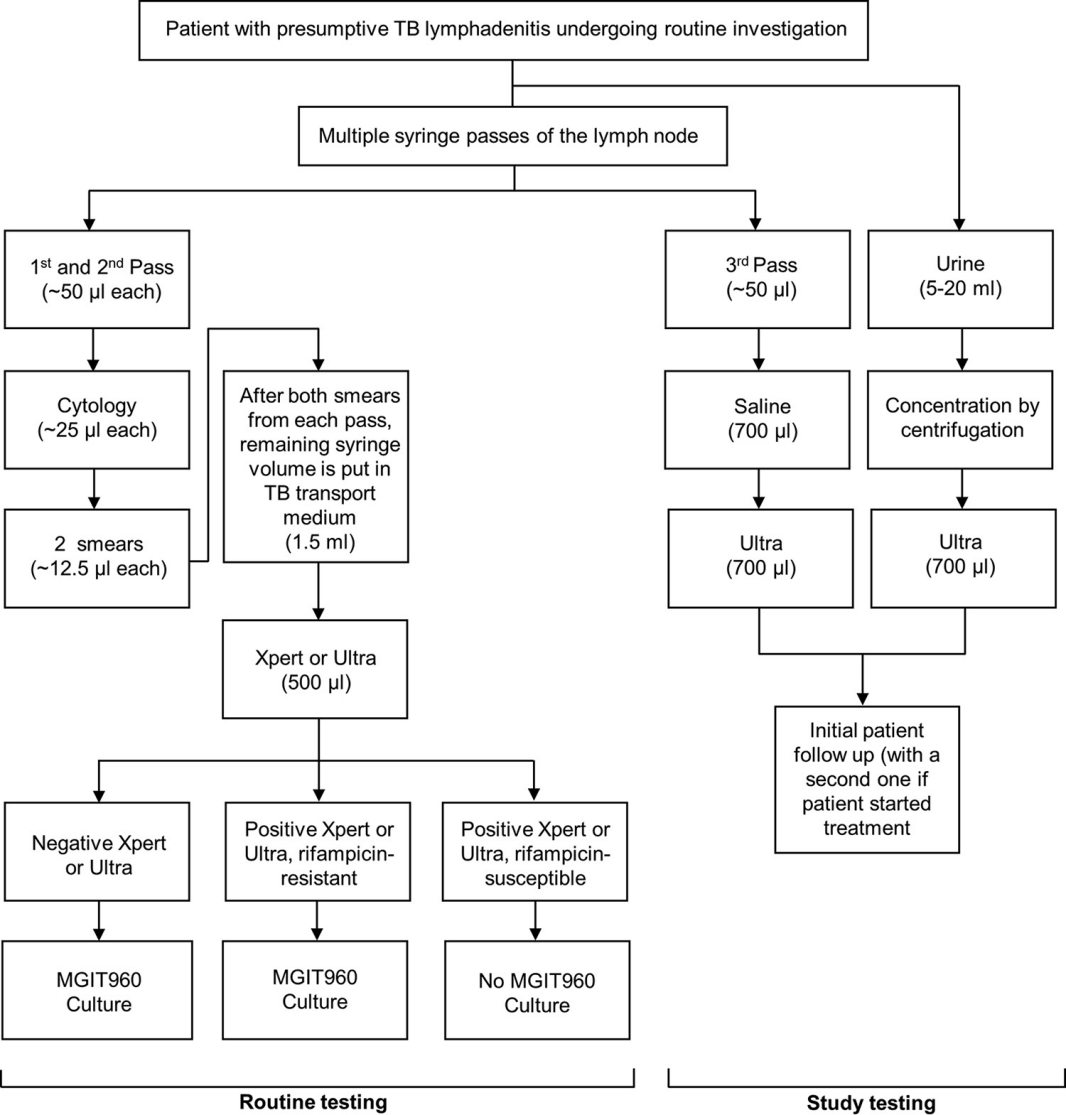
Specimen collection and diagnostic testing in participants with presumptive TB lymphadenitis. Abbreviations: TB, tuberculosis; Ultra, Xpert MTB/RIF Ultra; Xpert, Xpert MTB/RIF.

FNABs were collected by multiple needle passes using a 23-gauge needle and a 10-ml syringe. While the needle was inserted, negative suction with a cutting motion was applied for aspiration. The first two passes were used for routine cytology. From each pass, two slides were prepared: the first was air dried for Rapidiff staining, and the second was spray fixed for Papanicolaou staining (∼25 μl total volume used per pass) (Fig. 1). The remaining syringe contents were flushed into 1.5 ml TB transport medium (16). The third pass (5 to 50 μl) was collected into 700 μl 5% saline (Ysterplaat Medical Supplies, Cape Town, South Africa).

### Xpert, Ultra, and culture. (i) Routine testing.

Xpert (version 1; Cepheid, USA) was performed programmatically from 25 January 2017 to 9 April 2018 by the government programmatic laboratory (National Health Laboratory Service [NHLS]), who performed Ultra (version 1) thereafter (17). Sample reagent (2 ml; Cepheid, USA) was added to 500 μl of aspirate-containing 1.5 ml TB transport medium (4:1 ratio), and 2 ml of the mixture was used for Xpert or Ultra (18, 19). Per the algorithm, if a specimen was Xpert or Ultra positive and rifampicin susceptible, culture was not done. If a specimen was Xpert or Ultra negative or was Xpert or Ultra positive and rifampicin resistant, 500 μl aspirate-containing TB transport medium was inoculated into a MGIT 960 liquid culture without NALC-NaOH decontamination (Fig. 1). If a nonactionable (not positive or negative) (14) Xpert or Ultra result occurred, the remaining 500 μl TB transport medium was used to repeat the test.

### (ii) Study testing.

The third pass in 700 μl saline was tested with Ultra (cartridge version 3; study Ultra) using a 2:1 sample-to-reagent ratio (19). Study Ultra was done irrespective of whether routine Xpert or Ultra was done.

### (iii) MTBC typing and drug susceptibility testing.

The MTBDRplus assay was done on culture-positive isolates for species identification and drug susceptibility testing.

### Urine Ultra testing.

Five to 20 ml urine stored at −80°C was centrifuged (1,811 × *g*, 10 min, room temperature), and the supernatant was removed until 700 μl remained, which was tested with Ultra (2:1 sample-to-reagent volume ratio) (19).

### Patient treatment and follow-up.

Treatment decisions were programmatic without study involvement (no study results reported for patient management). Attempts were made to telephonically follow up patients at least 12 weeks after recruitment, at which point TB treatment initiation status was recorded and, if treatment started, treatment response was queried. Patients were lost to follow-up if at least two calls were unsuccessful and messages were unreturned for each time point.

### Definitions. (i) Patient groups.

Patients were designated definite, probable, or non-TB by use of different reference standards. For the microbiological reference standard (MRS), definite TB patients were culture positive and/or cytology positive on FNABs, and non-TB patients were culture and cytology negative on FNABs. Unclassifiable patients had no positive MRS test, culture was contaminated or not done, and cytology was not done. Table S1 in the supplemental material shows the extended microbiological standard (eMRS), composite reference standard (CRS), and probable TB definitions.

### (ii) Other definitions.

Xpert or Ultra actionable results for TB were MTBC detected and rifampicin susceptible, rifampicin resistant or rifampicin indeterminate, or MTBC not detected (14). For culture, actionable results were positive or negative for MTBC. For cytology, the presence or absence of granulomatous inflammation was recorded.

### Statistical analysis.

We included patients in head-to-head analyses if they had actionable routine index test (Xpert or Ultra), study Ultra, and culture results (or, if culture was nonactionable, a cytology result was available). Proportion tests (20) were done using STATA version 16.0 (StataCorp, College Station TX, USA) and GraphPad Prism version 8.0.1 (GraphPad Software, San Diego, USA). Venn diagrams were made with InteractiVenn (21). Differences in diagnostic accuracy metrics were calculated using proportion tests or McNemar’s test as appropriate. STARD guidelines were followed (22). We excluded the probable TB patients from the primary analysis due to few patients meeting this definition.

## RESULTS

### Patient characteristics.

Of 135 patients, 44% (59/135) were definite TB and 56% (75/135) were non-TB per the MRS. Characteristics are compared in Table 1.

**TABLE 1:** Demographic and clinical characteristics by microbiological reference standard status^*a*^

Demographic	Overall value (*n* = 135)	Value for patient group	
		Definite TB (*n* = 59)	Non-TB (*n* = 75)
Age (yrs)	36 (29–46.5)	34 (27–41)	39 (31.5–47.5) ***P* = 0.019**
Females	72/135 (53)	30/59 (51)	42/75 (56) *P* = 0.553

Clinical characteristics			
HIV positive	77/133 (58)	35/58 (60)	41/74 (55) *P* = 0.569
CD4 count (cells/μl) (range)	183 (66–304)	147 (43–281)	219 (156–358) ***P* = 0.012**
Previous TB	42/135 (31)	19/59 (32)	22/75 (29) *P* = 0.720
Pulmonary TB	38/42 (90)	17/59 (29)	20/75 (27) *P* = 0.783
Extrapulmonary TB	4/42 (10)	2/59 (3)	2/75 (3) *P* = 0.807
			
Involved site			
Neck	92/134 (67)	53/59 (90)	39/75 (52) ***P* < 0.001**
Thorax	16/134 (12)	4/59 (7)	12/75 (16) *P* = 0.102
Breast	9/134 (7)	0/59 (0)	9/75 (12) ***P* = 0.006**
Other^*b*^	17/134 (13)	2/59 (3)	15/75 (20) ***P* = 0.004**

a Definite TB patients were more likely to be younger, have an involved neck or breast lymph node (versus another anatomical site), and, if HIV positive, a lower CD4 count than non-TB patients. Data are number (%) or median (IQR). Missing data: HIV, two; CD4, four; lymph node site, one. One patient was unclassifiable based on case definitions. Boldface *P* values indicate significant differences (<0.05).

b Other sites included arm (*n* = 3), leg (*n* = 3), groin (*n* = 7), and head (*n* = 4).

### FNAB index test results.

**FIG 2:**
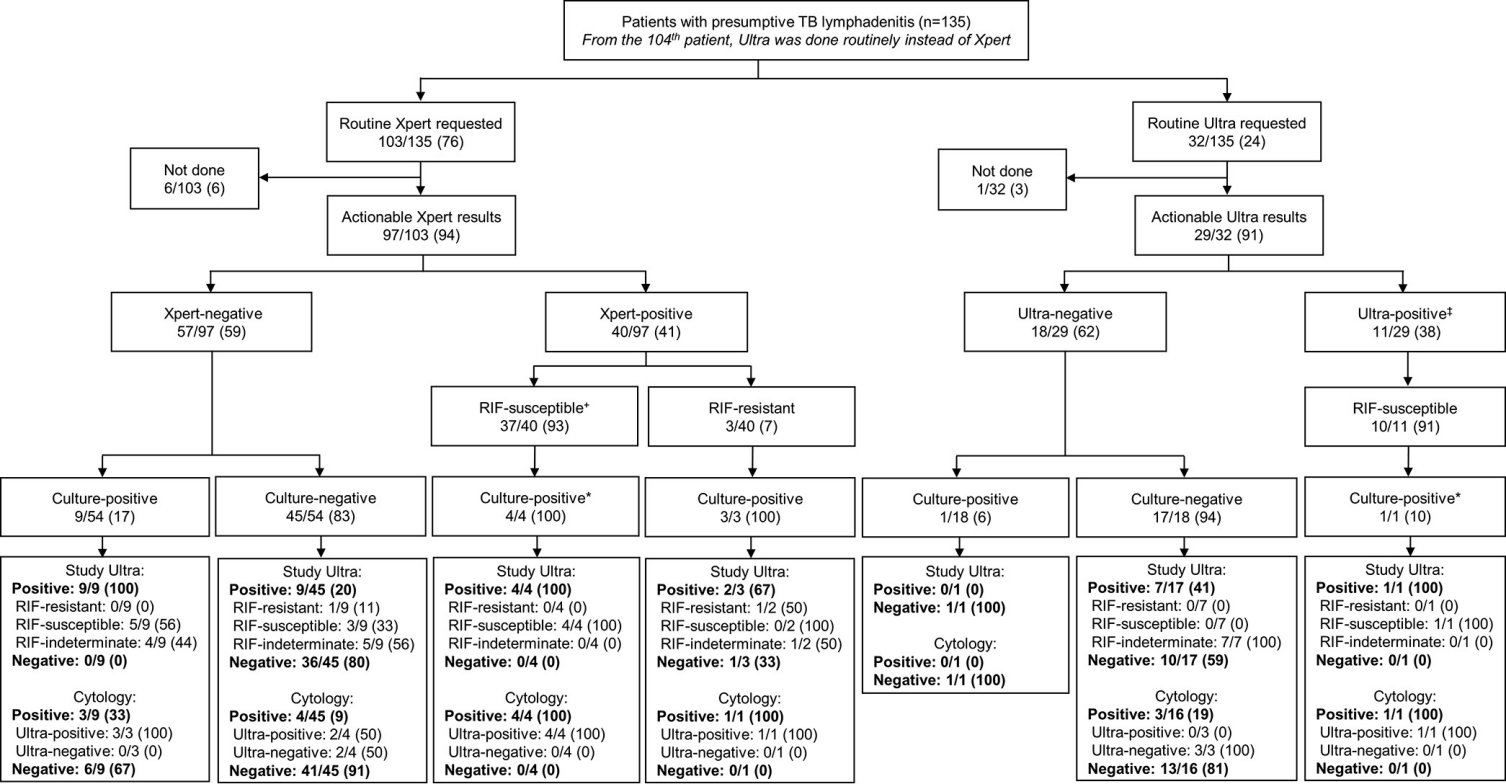
Overview of different FNAB-based test results. Tests done as part of the routine diagnostic algorithm (Xpert later replaced by Ultra, cytology, and culture) and the study (Ultra) are shown. Study Ultra detected TB in most culture-positive FNABs and some culture-negative FNABs. Italicized text indicates programmatic testing (programmatic algorithm adherence imperfect). Data are number of patients with that characteristic/total number of patients (*n*/*N*) (%). Abbreviations: RIF, rifampicin; TB, tuberculosis; Ultra, Xpert MTB/RIF Ultra; Xpert, Xpert MTB/RIF. Superscript symbols indicate the following. ^+^One routine Xpert-positive, rifampicin (RIF)-susceptible patient had a contaminated culture but was study Ultra positive and RIF resistant, and 32 routine Xpert-positive, rifampicin (RIF)-susceptible patients had no culture per the Fig. 1 algorithm. ^‡^One routine Ultra result was trace positive, RIF indeterminate. *Culture would not normally be requested per the routine algorithm for these patients but was nevertheless done. Ultras under “Cytology” subheadings (in the last row of boxes) are routine and not study Ultras. Missing data include the following. In patients with a routine Xpert-negative result, one had a contaminated culture and no culture was done for two. Two routine Ultra results were nonactionable. No cytology was done for three FNABs.

**FIG 3:**
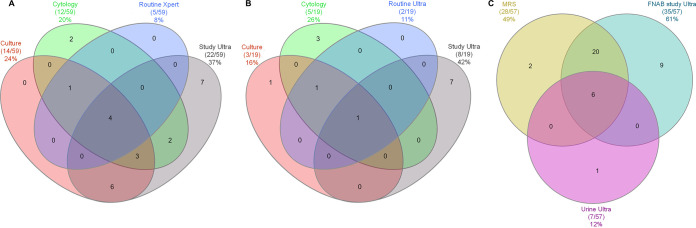
Venn diagrams showing positive results from different FNAB tests (after the 104th participant, Ultra was routinely done instead of Xpert) and urine Ultra. (A) Study Ultra, routine Xpert, culture, and cytology results in 59 patients. Study Ultra was positive in seven FNABs undetected by routine Xpert. (B) Routine Ultra results relative to study Ultra, routine Ultra, culture, and cytology in 19 patients. Study Ultra was exclusively positive in 36% (7/19) FNABs not detected by routine Ultra, culture, and cytology and had the highest yield. (C) Urine Ultra results relative to FNAB study Ultra and the MRS in 57 HIV-positive patients (urine Ultra negative in all HIV-negative patients). Urine Ultra detects less TBL than FNAB study Ultra but could obviate TB diagnostic FNABs in some patients. Data are number of patients positive/total number of patients (%). Abbreviations: FNAB, fine-needle aspirate; MRS, microbiological reference standard; TB, tuberculosis; Ultra, Xpert MTB/RIF Ultra; Xpert, Xpert MTB/RIF.

Seventy-six percent (103/135) of patients had routine Xpert requested (6/103 [6%] not done), and 24% (32/135) had routine Ultra requested (3% [1/32] not done). Nonactionable results for routine Xpert, routine Ultra, and study Ultra were 0% (0/97), 6% (2/31), and 3% (4/135), respectively. Forty-one percent (40/97) of routine Xpert results were positive (the remainder were negative). For routine Ultra results, 38% (11/29) were positive, and for study Ultra, 74/131 were positive (56%; *P* = 0.070 versus routine Ultra) (Fig. 2). In a head-to-head comparison of patients with actionable results from each test, i.e., study Ultra, routine Xpert, culture, and cytology, 37% (22/59), 8% (5/59), 20% (12/59), and 24% (14/59), respectively, were positive by each test (Fig. 3A; study Ultra had the highest yield). Twelve percent (7/59) of these patients with at least one positive result were exclusively detected by study Ultra (cytology exclusively detected two). This proportion detected only by study Ultra (and hence negative by routine Xpert and/or cytology) increased to 22% (13/59) when culture results, which are not available for rapid clinical decision making, were omitted.

### Diagnostic accuracy and yield of study Ultra and routine Xpert on FNABs. (i) Overall.

When Ultra was compared head-to-head to Xpert using the MRS (*n* = 92) (Table 2), Ultra had improved sensitivity (91% [95% confidence interval: 79, 98] versus 72% [57, 85]; *P* = 0.016) and decreased specificity (76% [61, 87] versus 93% [82, 99]; *P* = 0.020). Ultra’s positive predictive value (PPV) (79% [66, 89] versus 92% [78, 98]; *P* = 0.114) and negative predictive value (NPV) were like Xpert’s (90% [76, 97] versus 77% [64, 87]; *P* = 0.105). Conclusions were unchanged for non-head-to-head comparisons or those that used the eMRS or CRS (Table 2; see supplementary results in the supplemental material), which included patients with probable TB (Table S2). Compared to MTBDR*plus* on isolates, no false-negative or false-positive Ultra rifampicin resistance results occurred; however, numbers were small, precluding precise accuracy estimates (see supplementary results in the supplemental material).

**TABLE 2:** Diagnostic accuracy analyses (non-head-to-head and head-to-head) of routine Xpert and study Ultra on FNABs using an MRS (culture and cytology) for M. tuberculosis complex DNA detection stratified by HIV status^*a*^

Analysis and test	All patients (*n* = 96)				HIV negatives (*n* = 36/96 [38])				HIV positives (*n* = 60/96 [62])			
	Sensitivity	Specificity	PPV	NPV	Sensitivity	Specificity	PPV	NPV	Sensitivity	Specificity	PPV	NPV
Non-head-to-head^*b*^												
Xpert	73 (58, 85), 35/48	92 (80, 98), 44/48	90 (76, 97), 35/39	77 (64, 87), 44/57	65 (38, 86), 11/17	89 (67, 99), 17/19	85 (55, 98), 11/13	74 (52, 90), 17/23	77 (59, 90), 24/31 *P* = 0.343*	93 (77, 99), 27/29 *P* = 0.656*	92 (75, 99), 24/26 *P* = 0.455*	79 (62, 91), 27/34 *P* = 0.627*
	*n* = 130				*n* = 55/128 (43)				*n* = 73/128 (47)			
Ultra	85 (73, 93), 51/60 *P* = 0.121^‡^	69 (56, 79), 48/70 ***P* = 0.003**^‡^	70 (58, 80), 51/73 ***P* = 0.018**^‡^	84 (72, 93), 48/57 *P* = 0.343^‡^	76 (55, 91), 19/25 *P* = 0.427^‡^	70 (51, 85), 21/30 *P* = 0.111^‡^	68 (48, 84), 19/28 *P* = 0.260^‡^	78 (58, 91), 21/27 *P* = 0.750^‡^	91 (76, 98), 31/34 *P* = 0.125^‡^ *P* = 0.109*	67 (50, 81), 26/39 ***P* = 0.009**^‡^ *P* = 0.768*	70 (55, 83), 31/44 ***P* = 0.031**^‡^ *P* = 0.816*	90 (73, 98), 26/29 *P* = 0.267^‡^ *P* = 0.227*
Change if traces excluded	−2 (−15, 12) *P* = 0.808^§^	+15 (1, 30) ***P* = 0.041**^§^	+13 (−1, 28) *P* = 0.081^§^	0 (−13, 13) *P* > 0.999^§^	−3 (−28, 22) *P* = 0.797^§^	+21 (1, 41) *P* = 0.076^§^	+21 (−2, 44) *P* = 0.103^§^	0 (−22, 22) *P* > 0.999^§^	−1 (−15, 13) *P* = 0.905^§^	+12 (−8, 32) *P* = 0.253^§^	+10 (−9, 28) *P* = 0.332^§^	0 (−16, 16) *P* > 0.999^§^
Change if traces reclassified	−10 (−19, −1) ***P* = 0.014**^§^	+18 (8, 29) ***P* < 0.001**^§^	+13 (−1, 28) *P* = 0.081^§^	−4 (−17, 9) *P* = 0.558^§^	−12 (−29, 5) *P* = 0.083^§^	+23 (5, 42) ***P* = 0.008**^§^	+21 (−2, 44) *P* = 0.103^§^	−2 (−23, 18) *P* = 0.845^§^	−9 (−21, 4) *P* = 0.083^§^	+15 (1, 29) ***P* = 0.014**^§^	+10 (−9, 28) *P* = 0.332^§^	−6 (−21, 11) *P* = 0.517^§^
	
Head-to-head	*n* = 92				*n* = 35/92 (38)				*n* = 57/92 (62)			
Xpert	72 (57, 84), 33/46	93 (82, 99), 43/46	92 (78, 98), 33/36	77 (64, 87), 43/56	65 (38, 86), 11/17	94 (73, 100), 17/18	92 (62, 100), 11/12	74 (52, 90), 17/23	76 (56, 96), 22/29 *P* = 0.417*	93 (76, 99), 26/28 *P* = 0.832*	92 (73, 99), 22/24 *P* > 0.999*	79 (61, 91), 26/33 *P* = 0.671*
Ultra	91 (79, 98), 42/46 ***P* = 0.016**^‡^	76 (61, 87), 35/46 ***P* = 0.020**^‡^	79 (66, 89), 42/53 *P* = 0.114^‡^	90 (76, 97), 35/39 *P* = 0.105^‡^	82 (57, 96), 14/17 *P* = 0.244^‡^	72 (47, 90), 13/18 *P* = 0.074^‡^	74 (49, 91), 14/19 *P* = 0.217^‡^	81 (54, 96), 13/16 *P* = 0.593^‡^	97 (82, 100), 28/29 ***P* = 0.022**^‡^ *P* = 0.099*	79 (59, 92), 22/28 *P* = 0.127^‡^ *P* = 0.622*	82 (65, 93), 28/34 *P* = 0.311^‡^ *P* = 0.455*	96 (78, 100), 22/23 *P* = 0.076^‡^ *P* = 0.145*
Change if traces excluded	−1 (−17, 11) *P* = 0.836^§^	+7 (−9, 24) *P* = 0.400^§^	+5 (−11, 20) *P* = 0.576^§^	0 (−13, 13) *P* > 0.999^§^	−3 (=32, 24) *P* = 0.791^§^	+14 (−12, 41) *P* = 0.321^§^	+11 (−17, 39) *P* = 0.463^§^	0 (−27, 27) *P* > 0.999^§^	1 (−10, 10) *P* = 0.937^§^	+3 (−18, 24) *P* = 0.787^§^	+1 (−18, 19) *P* = 0.917^§^	0 (−12, 12) *P* > 0.999^§^
Change if traces reclassified	−13 (−25, 1) ***P* = 0.014**^§^	+9 (−2, 19) ***P* = 0.046**^§^	+5 (−11, 20) *P* = 0.576^§^	−10 (−25, 5) *P* = 0.196^§^	−17 (−42, 6) *P* = 0.083^§^	+17 (−6, 39) *P* = 0.083^§^	+11 (−17, 39) *P* = 0.462^§^	−8 (−35, 18) *P* = 0.542^§^	−11 (−25, 4) *P* = 0.083^§^	+3 (−7, 14) *P* = 0.317^§^	+1 (−18, 19) *P* = 0.917^§^	−11 (−26, 5) *P* = 0.219^§^

a Study Ultra has improved sensitivity relative to routine Xpert but lower specificity. The relative performances of Xpert and Ultra had similar patterns by HIV status and in comparison to the eMRS or CRS (see Table S2 in the supplemental material). Data are presented as % (95% CI), number of patients/total number. Within-column *P* values: ^‡^Xpert versus Ultra within an analysis (non-head-to-head, head-to-head) in patients of the same HIV status (overall, negative, positive). ^§^Xpert versus Ultra (either with traces excluded or with traces reclassified) within an analysis (non-head-to-head, head-to-head) in patients of the same HIV status (overall, negative, positive). Within-row *P* values: *HIV-negative patients versus HIV-positive patients within an analysis (non-head-to-head, head-to-head). Boldface *P* values indicate significant differences (<0.05). Abbreviations: CI, confidence interval; CRS, composite reference standard; eMRS, extended microbiological reference standard; FNABs, fine-needle aspirate biopsy specimens; MRS, microbiological reference standard; NPV, negative predictive value; PPV, positive predictive value; Ultra, Xpert MTB/RIF Ultra; Xpert, Xpert MTB/RIF.

b Missing data in the non-head-to-head table: unclassifiable Ultra, *n* = 1; nonactionable Ultras, *n* = 4; HIV, *n* = 2.

### (ii) HIV.

Sensitivities and specificities did not differ between HIV-positive and HIV-negative patients for study Ultra or routine Xpert (Table 2). Within HIV-positive patients, Ultra had improved sensitivity (97% [82, 100] versus 76% [56, 96]; *P* = 0.022) and similar specificity (79% [59, 92] versus 93% [76, 99]; *P* = 0.127) to Xpert.

### (iii) Trace semiquantitation exclusion or reclassification.

When study Ultra traces were excluded, sensitivity (−1% [−17, 11]; *P* = 0.836) and specificity (+7% [−9, 24]; *P* = 0.400) were unchanged. When trace results were reclassified as negative, sensitivity decreased (−13% [−25, 1], *P* = 0.014) and specificity increased (+9% [−2, 19], *P* = 0.046) (Table 2).

### (iv) Ultra PCR inhibition.

An analysis of sample processing control (SPC) threshold cycle (*C_T_*) values (see Fig. S1 in the supplemental material) (higher values indicate more inhibition) showed more inhibition in study Ultra positives than negatives (25.80 [interquartile range {IQR}, 24.78 to 27.33] versus 25.20 [24.55 to 26.05]; *P* = 0.024). Furthermore, false negatives were more inhibited than true positives (26.10 [25.10 to 28.60] versus 25.10 [24.00 to 25.50]; *P* = 0.001), suggesting that inhibition contributes to diminished sensitivity.

### (v) Relationship with bacterial load.

Neither study Ultra nor routine Xpert *C_T_* values correlated with bacillary load measured using culture time to positivity (Fig. S2) in FNABs.

### Comparison of study Ultra true positives and false positives.

False positives had less bacterial load than true positives (IS*6110/*IS*1081 C_T_*, 19.00 [IQR, 16.40 to 21.60) versus 24.85 [19.88 to 28.15]; *P* < 0.001), so a greater proportion were hence “trace” (59% [13/22] versus 12% [6/51]; *P* < 0.001) (Table 3). Less inhibition was also observed for the former group (SPC *C_T_*, 25.05 [24.45 to 25.95] versus 26.10 [25.10 to 28.60]; *P* = 0.005). More study Ultra true-positive patients were on treatment at follow-up than Ultra false positives (92% [44/48] versus 27% [6/22]; *P* < 0.001), as more true positives were positive using a routine test than the false positives (98% [50/51] versus 27% [6/22]; *P* < 0.001). The proportions of patients with previous TB in false positives versus true positives were similar (27% [6/22] versus 35% [18/51]; *P* = 0.503). The characteristics of true and false positives are shown in Table 3, and the false positives per patient information in shown in Table S3.

**TABLE 3:** Comparison of patient and microbiology characteristics by whether study Ultra result was true positive or false positive per the MRS^*a*^

Characteristic	Value for:	
	Ultra true positives	Ultra false positives

Patient characteristics		
HIV positive	31/51 (61)	13/22 (59) *P* = 0.892
CD4 count (cells/μl)	147.0 (32.00–281.30) (*n* = 30)	208.0 (101.3–286.0) (*n* = 12) *P* = 0.238
Previous TB	18/51 (35)	6/22 (27) *P* = 0.503
Patients initiated on TB treatment after 12-wk follow-up^*b*^	44/48 (92)	6/22 (27) ***P* < 0.001**
If on treatment, did the patient report improved health?	43/44 (98)	6/6 (100) *P* = 0.709

Study Ultra result information		
*rpoB C_T_*_min_	25.70 (20.20–28.20) (*n* = 45)	25.70 (20.40–29.10) (*n* = 9) *P* = 0.878
IS*6110/*IS*1081 C_T_*	19.00 (16.40–21.60) (*n* = 51)	24.85 (19.88-28.15) (*n* = 22) ***P* < 0.001**
Trace semiquantitation category	6/51 (12)	13/22 (59) ***P* < 0.001**
SPC *C_T_*	26.10 (25.10–28.60) (*n* = 51)	25.05 (24.45–24.95) (*n* = 22) ***P* = 0.005**

Routine Xpert or routine Ultra information		
Positive Xpert	31/42 (74)	3/11 (27) ***P* = 0.004**
Positive Ultra	7/7 (100)	3/10 (30) ***P* = 0.004**

a False-positive (FP) patients were less likely to have been placed on treatment, had less bacterial load, and were less likely to have been detected by routine Xpert and routine Ultra than true-positive (TP) patients. An individual breakdown of each Ultra-positive, MRS-negative patient is shown in Table S3 in the supplemental material. Data are number of patients/total number (%) or median (IQR). Missing data include the following: CD4 count, *n* = 2; patients who were lost to follow up, *n* = 3; unclassifiable routine Xpert results, *n* = 3. True positive in routine Xpert era not done, *n* = 1; true positive in routine Ultra era nonactionable, *n* = 1; false positive in routine Ultra not done, *n* = 1. Boldface *P* values indicate significant differences (<0.05). Abbreviations: IS*6110/*IS*1081 C_T_*, cycle threshold value for the Xpert MTB/RIF Ultra IS*6110/*IS*1081* probe; *rpoB C_T_*_min_, minimum cycle threshold value from the Xpert MTB/RIF (Ultra) *rpoB* probes; Ultra, Xpert MTB/RIF Ultra.

b Study Ultra results were not reported for potential patient management.

### Study versus routine Ultra FNAB results. (i) Concordance.

In patients who received both study and programmatic Ultra tests, 55% (17/31) were study Ultra positive and 35% (11/31) were routine Ultra positive. The former detected +20% (95% confidence interval. 0, 42) more TBL (Table 4).

**TABLE 4:** Study and routine Ultra concordance in patients with both tests done on FNABs^*a*^

Routine Ultra result	Study Ultra result (no. of patients)		Total no. of patients
	Positive	Negative	
Positive	10	1	11
Negative	7	11	18
Total	18	13	31
Nonactionable^*b*^	1	1	2
		
Change in study Ultra result vs routine Ultra result	+20% (95% CI: 0, 42) ***P* = 0.034**		

a More patients were positive by study Ultra (55%) than by routine Ultra (35%), corresponding to a 20% incremental yield. Study Ultra had no nonactionable results (data not shown). A boldface *P* value indicates a significant difference (<0.05). Abbreviations: Ultra, Xpert MTB/RIF Ultra; FNABs, fine-needle aspirate biopsy specimens; CI, confidence interval.

b Nonactionable Ultra results included “error” (*n* = 1) and “no result” (*n* = 1).

### (ii) PCR inhibition.

SPC *C_T_* analysis showed no difference between study and routine Ultra results (25.10 [IQR, 24.35 to 25.85] versus 25.50 [24.20 to 26.50]; *P* = 0.081) (Fig. S1A).

### Urine Ultra yield, sensitivity, and specificity and nonactionable results.

Urine Ultra testing had low sensitivity (18% [7, 35]) and high specificity (98% [88, 100]) (head-to-head comparisons with FNAB study Ultra are shown in Table S4). Of concentrated urine specimens tested (*n* = 84), 8% (7/84) were nonactionable and 100% (7/7) of these resolved to actionable when unconcentrated urine specimens were tested (one unconcentrated urine specimen was now Ultra positive). None of the 18 HIV-negative patients had any positive urine Ultra results. Twelve percent (7/57) of the HIV-positive patients were urine Ultra positive (six of seven detected by both positive MRS and study Ultra FNAB result) (Fig. 3C). In other words, when urine Ultra was attempted among HIV positives, 11% (7/64, 3 of which were trace) were positive, meaning that universal concentrated urine Ultra testing in HIV positives with presumptive TBL could reduce the number of FNABs required for TB diagnosis, as few are nonactionable.

### Patient treatment status at follow-up.

Ninety-six percent (130/135) of patients were followed up (median, 37 weeks [IQR, 16 to 65 weeks] since recruitment), and 52% (68/130) initiated treatment. Of the 48% (62/130) not initiated on treatment, 11% (7/62) were definite TB and 89% (55/62) were non-TB, of which 57% (4/7) and 29% (16/55) were study Ultra positive, respectively. In these definite-TB patients not on treatment but detected by study Ultra, 50% (2/4) were detected by routine Ultra or Xpert, meaning that study Ultra was the only rapid PCR test available in half of these cases (these two definite-TB patients were pretreatment lost to follow-up). Regarding the clinical status of patients initiated on treatment, 94% (64/68) reported treatment completion, and of these, 94% (60/64) reported being clinically well. Three percent of the patients followed up (4/130) died (one of these four was study Ultra positive [routine Ultra negative, Xpert negative]), and100% (4/4) of the decedents were non-TB and not placed on treatment.

## DISCUSSION

Our key findings are the following. (i) Study Ultra on FNABs had, in comparison to Xpert, improved sensitivity and decreased specificity and outperformed routine Ultra (tests unaffected by HIV, alternative reference standards, and probable TBs). (ii) Approximately 3 in 10 study Ultra positives had not been placed on treatment, indicating opportunities to improve TBL treatment with Ultra. (iii) Exclusion of study Ultra trace results improved specificity (more so than reclassifying to negative) without large sensitivity costs relative to treating Ultra trace results as positive. (iv) Urine Ultra testing had low sensitivity but could reduce the proportion of presumptive TBL patients who require an FNAB in our setting. (v) Ultra false-negative results are associated with PCR inhibition. These data show high sensitivity of Ultra on FNABs for TBL with the inclusion of trace-positive results (without which sensitivity benefits over Xpert are not seen).

Ultra on FNABs had higher sensitivity than Xpert, suggesting that Ultra is a rapid initial test for TBL. Ultra still did not detect, however, approximately 1 in 10 TBL cases, indicating a sustained need for more sensitive tests (especially those that use noninvasive specimens) and a continued role for reflex tests for downstream testing of Ultra-negative FNABs. Importantly, as was done previously for Xpert (23), we showed that one likely cause of Ultra false negativity is increased PCR inhibition (which could be caused by mucopurulent or viscous samples, as seen in sputum [23]), suggesting that optimized specimen processing workflows to better remove interfering agents are still needed to boost sensitivity.

Notably, Ultra had suboptimal specificity (2 in 10 MRS-negative people were study Ultra positive). One reason may be that culture and cytology have limitations as reference standards for EPTB (8). Notably, this finding mirrors prior work on TBL that used tissue in addition to fluid biopsy specimens for Ultra, where a specificity of 78% versus culture was observed (13). However, when compared to an eMRS that included microbiological tests such as FNAB culture as well as culture and Ultra on non-site-of-disease fluids, FNAB Ultra specificity was 100% in that study. In contrast, we applied microbiological tests only to FNABs and did not exhaustively sample anatomical sites (24), which might underestimate specificity.

Ultra false positivity was more frequent in patients with less mycobacterial DNA, and in contrast to pulmonary TB, FNAB Ultra false positivity was not associated with prior TB (14). The true nature of these Ultra “false positives” in EPTB requires clarification and is an important topic for future research (in our setting, most “false-positive” patients with presumptive pulmonary TB remain well without treatment) (25, 26). Such “false-positive” results could be caused by M. tuberculosis in FNABs that is not culturable using conventional methods like MGIT 960. For example, in animal models, M. tuberculosis DNA in lymph nodes is detectable during reactivation of TB, despite no pathological evidence of disease and no culturability. M. tuberculosis is hypothesized to then disseminate throughout the body from the lymph nodes (27). Moreover, we observed no correlation in bacterial load measured between Ultra and culture, further supporting the presence of M. tuberculosis DNA in the absence of culturability.

Critically, if Ultra trace results were excluded or reclassified to elevate specificity, Ultra would lose sensitivity benefits in comparison to Xpert; however, this sensitivity loss was less for the former than the latter strategy, suggesting that exclusion is the preferred strategy for handling trace results.

When routine and study Ultra concordances were analyzed, study Ultras had a higher yield. This may be due to specimen processing (e.g., more sample reagent is used for routine Ultras than for study Ultras) or cartridge version differences (which includes extending product stability and improving product manufacturability without affecting assay performance) but is overall indicative of an area to improve the diagnosis of TBL within the program.

Few studies examined Ultra on urine specimens (28–30) and none in patients investigated for TBL. Urine Ultra may obviate invasive sampling (and hence referral to a specialized facility and associated costs and delays). Despite concentration (31), low yield and sensitivity were observed for urine Ultra, suggesting it could marginally reduce FNAB collection (approximately 1/10). Such a strategy is undermined by elevated nonactionable result rates and cost effectiveness, including the number needed to test, and would require prospective investigation and modeling; however, we expect the utility of such an approach to be further enhanced with better urine tests (we did not have access to Fujifilm SILVAMP) (32).

These results have strengths and limitations. Our study was pragmatic, routine culture was not always done, and although our MRS included cytology, multiple cultures (including on specimens from other anatomical sites) may improve specificity estimates. Furthermore, multiple FNAB passes were done to obtain adequate volumes that could have introduced sampling variation; however, FNABs were collected using a standardized protocol by a single health care worker. Additionally, a third of the FNABs collected for routine testing were used for Ultra and had more sample reagent (SR) buffer added for testing (a 4:1 ratio of SR buffer to sample, the programmatic standard of care) than that used for study Ultras, in which the full sample collected was used and a 2:1 ratio of SR buffer was added. A different cartridge version (version 1) was used in routine testing, whereas study Ultra used a later version (version 3); however, in an internal head-to-head version evaluation done by the programmatic laboratory (as part of separate study), no sensitivity differences between different versions were observed (most changes between versions were done to improve stability and optimize manufacturing), meaning that it is unlikely that these version differences in routine versus study Ultra accounted for any meaningful performance differences. Thus, the differences observed primarily appear to be due to sampling and specimen processing differences, which improved sensitivity and yield; however, our study was not designed to quantify the contribution of each component that differed between routine and study Ultra testing.

In conclusion, in a routine clinical setting in patients with presumptive TBL, Ultra detects more TBL than Xpert and would result in more people being placed on treatment. This is driven by the added benefit of trace results. Furthermore, programmatic Ultra testing can be optimized on the diagnostic laboratory front, as study Ultra had better performance than that done routinely. Urine Ultra testing could reduce invasive sampling and associated delays, but there remains a need for better urine-based tests for TBL. We recommend that a positive FNAB Ultra result be used to initiate treatment; however, patients with a negative Ultra result still require confirmatory testing, and many patients with a trace-positive result will be culture negative. Our study supports Ultra’s use for TBL diagnosis.
