# Supplementary material for: Xpert MTB/RIF Ultra Is Highly Sensitive for the Diagnosis of Tuberculosis Lymphadenitis in a High-HIV Setting
Source: J Clin Microbiol. 2021 Nov 18;59(12):e01316-21. doi: 10.1128/JCM.01316-21 (PMC8601227; doi:10.1128/JCM.01316-21)
Supplement: Supplemental file 1 — Supplemental text, Tables S1 to S8, and Fig. S1 and S2. Download jcm.01316-21-s0001.pdf, PDF file, 0.4 MB [file jcm.01316-21-s0001.pdf]

Table of Contents

Supplementary Table 1: Reference standard definitions 2

Definitions 3

Microbiological reference standard 3

Extended microbiological reference standard 3

Composite reference standard 3

Supplementary Results 5

Bacterial load in study Ultra and routine Xpert 5

Drug susceptibility results of study Ultras on FNABs 5

Supplementary Figure 1: Spaghetti and box and whiskers plots showing FNAB Ultra internal positive control quantitative information (SPC CT). (A) Study SPC CTs vs. routine SPC CTs 6

Supplementary Figure 2: Quantitative information of Ultra (IS6110/IS1081, rpoB) and Xpert (rpoB) compared with bacillary load (MGIT960 liquid culture TTP) 7

Supplementary Table 2: Non-head-to-head and head-to-head diagnostic accuracy analyses of Xpert and Ultra using a microbiological reference standard (MRS), extended microbiological reference standard (eMRS) and composite reference standard (CRS) for the detection of Mycobacterium tuberculosis complex DNA 8

Supplementary Table 3: Study Ultra-positive patients who were culture- and cytology-negative, with information on their Ultra semi-quantitation category, previous TB status, TB treatment initiation status and patient’s status after at least 12-weeks of follow-up 9

Supplementary Table 4: Diagnostic accuracy of Ultra on urine measured using the microbiological reference standard (MRS) in a head-to-head analysis stratified by HIV status 11

Supplementary Table 5: Diagnostic accuracy analyses (non-head-to-head top, head-to-head bottom) of routine Xpert and study Ultra (excluding traces) on FNABs using a MRS stratified by HIV status **Error! Bookmark not defined.**

Supplementary Table 6: Non-head-to-head and head-to-head diagnostic accuracy analyses of Xpert and Ultra using a microbiological reference standard (MRS), extended microbiological reference standard (eMRS) and composite reference standard (CRS) for the detection of Mycobacterium tuberculosis complex DNA, excluding trace positive Ultra results 12

Supplementary Table 7: Diagnostic accuracy analyses (non-head-to-head top, head-to-head bottom) of routine Xpert and study Ultra (reclassifying traces as negative) on FNABs using a MRS stratified by HIV status **Error! Bookmark not defined.**

Supplementary Table 8: Non-head-to-head and head-to-head diagnostic accuracy analyses of Xpert and Ultra using a microbiological reference standard (MRS), extended microbiological reference standard (eMRS) and composite reference standard (CRS) for

the detection of Mycobacterium tuberculosis complex DNA, reclassifying trace positive Ultra results as negative 14

Supplementary Table 1: Reference standard definitions

	MRS[*]	eMRS[†]	CRS[‡]
	**Site of disease fluid**		
Xpert	✘	✓	✓
MGIT960 Culture	✓	✓	✓
Cytology	✓	✓	✓
	**Non-site-of disease fluid**		
Smear	✘	✓	✓
Xpert	✘	✓	
Ultra	✘	✓	✓
MGIT960 Culture	✘	✓	✓
	**Treatment information**		
TB treatment initiated	✘	✘	✓
Response to treatment self-reported by patient	✘	✘	✓
	**Case definitions**		
Reference standard **positive (Definite TB cases)**	Any MRS test positive	Any eMRS test positive	Any eMRS test positive/or TB treatment was initiated and response to treatment documented
Reference standard **negative (Non-TB patients)**	No MRS test positive	No eMRS test positive	No eMRS test positive and patient not initiated on treatment
**Probable TB patients**	N/A	N/A	No eMRS test positive, but treatment initiated
**Unclassifiable**	No positive MRS test and site-of-disease fluid culture contaminated or not done	No positive eMRS test and site-of-disease fluid culture contaminated or not done	No positive eMRS test and site-of-disease fluid culture contaminated or not done, or treatment not initiated

Abbreviations: Composite reference standard, CRS; Extended reference standard, eMRS; MGIT960 culture, Mycobacteria Growth Indicator Tube 960; Microbiological reference standard, MRS; Smear, smear microscopy; Ultra, Xpert MTB/RIF Ultra; Xpert, Xpert MTB/RIF.

Definitions

Microbiological reference standard

For the microbiological reference standard (MRS), a definite TB case was defined as a fine needle aspirate (FNAB) being culture-positive or cytology-positive and a non-TB patient was defined as being FNAB culture and cytology negative. Patients were unclassifiable if they had no positive MRS test and the site-of-disease culture was either contaminated or not done or cytology was not done.

Extended microbiological reference standard

For the extended microbiological reference standard (eMRS), a definite TB case was defined as a FNAB or any other body fluid being culture-, smear-, routine Xpert- or Ultra- positive and a non-TB case was defined as FNAB and other body fluids being culture-, smear-, Xpert-and Ultra- negative. Patients were considered unclassifiable if they had no positive eMRS test and the site-of-disease culture was either contaminated or not done.

Composite reference standard

For the composite reference standard (CRS), a definite TB case was defined as a FNAB or any other body fluid being culture-, smear-, Xpert- or Ultra- positive or TB treatment was initiated and response to treatment is documented; a probable-TB case was defined as a FNAB or any other body fluid being culture-, smear-, Xpert- or Ultra- positive or the patient being initiated on TB treatment after the 12-week follow up; and a non-TB case was defined as FNAB and other body fluids being culture-, smear-, Xpert-and Ultra- negative, and the patient was not initiated on TB treatment, and the patient was diagnosed with an alternative disease. Patients were unclassifiable if they had no positive eMRS test and the site-of-disease culture was either contaminated or not done, and treatment was not initiated.

Supplementary Results

Bacterial load in study Ultra and routine Xpert

No correlations were observed between study Ultra quantitation (IS*6110*/IS*1081* C_T and *rpoB* C_{Tmin}) and culture time-to-positivity (TTP) and routine Xpert quantitation (*rpoB* C_{Tmin}) and culture TTP (**Supplementary Figure 2**).

Drug susceptibility results of study Ultras on FNABs

Of 74 study Ultra-positive patients, 70% (52/74) were rifampicin-susceptible, 4% (3/74) resistant, and 26% (19/74) indeterminate (all trace). In patients who had actionable study Ultra and culture results (n=84), 20% (17/84) had MTBDR*plus* done. Of these, 18% (3/17) were MTBDR*plus* rifampicin-resistant and 82% (14/17) susceptible. 33% (1/3) of these MTBDR*plus* rifampicin-resistant patients were study Ultra rifampicin-resistant (one study Ultra trace-positive, rifampicin indeterminate and the other study Ultra negative), and 57% (8/14) of MTBDR*plus* rifampicin-susceptible patients were study Ultra rifampicin-susceptible (the remaining four were study Ultra trace-positive, rifampicin indeterminate and other remaining two were study Ultra-negative).

Supplementary Figure 1: Spaghetti and box and whiskers plots showing FNAB Ultra SPC C_T. **(A)** Study SPC C_T vs. routine SPC C_T. (**B**) SPC C_T from positive and negative study Ultras. More inhibition was observed in positives. (**C**) SPC C_T in true-positive vs. false-negative study Ultras, showing that greater inhibition is associated with Ultra missing TBL cases. Abbreviations: FNAB, fine needle aspirate biopsy; SPC C_T; sample processing control cycle threshold value for the Xpert MTB/RIF Ultra (Ultra) internal positive control which measures PCR inhibition; Ultra, Xpert MTB/RIF Ultra.

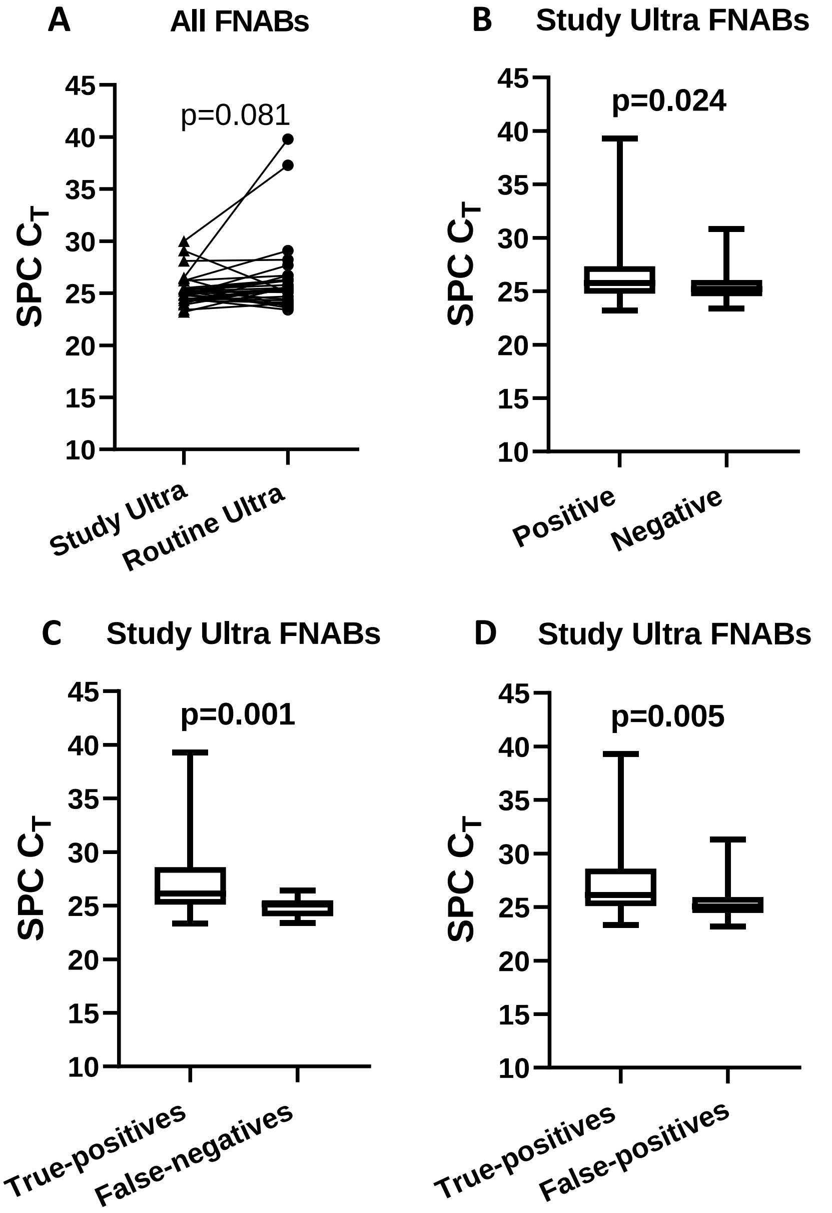

Supplementary Figure 2: FNAB Quantitative information of Ultra (IS*6110*/IS*1081*, *rpo*B) and Xpert (*rpo*B) compared with bacillary load (MGIT960 liquid culture TTP)**. (A**) Study Ultra IS*6110*/IS*1081* C_T vs. MGIT960 liquid culture TTP. (B) Study Ultra *rpo*B C_{Tmin} vs. MGIT960 liquid culture TTP**. (C)** Routine Xpert *rpo*B $C_T$min vs. MGIT960 liquid culture TTP. No correlations were observed. Only two culture-positive, routine Ultra-positive FNABs were present and routine Ultra results are hence not graphed. Abbreviations: FNAB, fine needle aspirate biopsy; TTP, culture time-to-positivity; Ultra, Xpert MTB/RIF Ultra; Xpert, Xpert MTB/RIF.

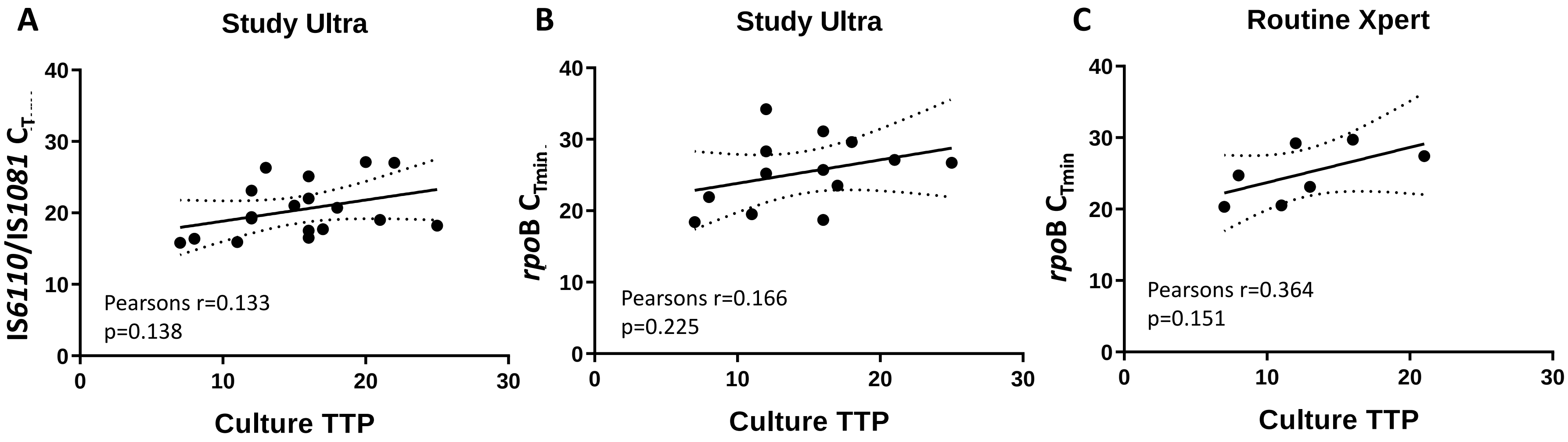

Supplementary Table 2: Non-head-to-head and head-to-head diagnostic accuracy analyses of Xpert and Ultra using a MRS, eMRS and CRS for the detection of Mycobacterium tuberculosis complex DNA. Conclusions were like those for the MRS **(Table 2)**. Data are %, 95% CI, and n/N

	**Non-head-to-head**											
	**MRS**				**eMRS**				**CRS**			
	**n=96**				**n=97**				**n=97**			
	**Sensitivity**	**Specificity**	**PPV**	**NPV**	**Sensitivity**	**Specificity**	**PPV**	**NPV**	**Sensitivity**	**Specificity**	**PPV**	**NPV**
**Xpert**	73 (58, 85)<br>35/48	92 (80, 98)<br>44/48	90 (76, 97)<br>35/39	77 (64, 87)<br>44/57	69 (55, 81)<br>36/52<br>p=0.685[*]	91 (79, 98)<br>41/45<br>p=0.924[*]	90 (76, 97)<br>36/40<br>p=0.970[*]	72 (58, 83)<br>41/57<br>p=0.519[*]	65 (52, 77)<br>39/60<br>p=0.635[±]<br>p=0.379[¥]	97 (86, 100)<br>36/37<br>p=0.244[±]<br>p=0.274[¥]	98 (87, 100)<br>39/40<br>p=0.166[±]<br>p=0.157[¥]	63 (49, 76)<br>36/57<br>p=0.317[±]<br>p=0.102[¥]
	**n=130**				**n=131**				**n=131**			
**Ultra**	85 (73, 93)<br>51/60<br>p=0.121[‡]	69 (56, 79)<br>48/70<br>**p=0.003**[‡]	70 (58, 80)<br>51/73<br>**p=0.018**[‡]	84 (76, 97)<br>48/57<br>p=0.343[‡]	83 (71, 91)<br>53/64<br>p=0.085[‡]<br>p=0.741[*]	69 (56, 79)<br>46/67<br>**p=0.005**[‡]<br>p=0.991[*]	72 (60, 81)<br>53/74<br>**p=0.024**[‡]<br>p=0.815[*]	81 (68, 90)<br>46/57<br>p=0.271[‡]<br>p=0.622[*]	76 (65, 85)<br>58/76<br>p=0.147[‡]<br>p=0.345[±]<br>p=0.207[¥]	71 (57, 82)<br>39/55<br>**p=0.001**[‡]<br>p=0.788[±]<br>p=0.778[¥]	78 (67, 87)<br>58/74<br>**p=0.006**[‡]<br>p=0.343[±]<br>p=0.238[¥]	68 (55, 80)<br>39/57<br>p=0.554[‡]<br>p=0.132[±]<br>**p=0.047**[¥]
	**Head-to-head**											
	**n=92**				**n=92**				**n=92**			
**Xpert**	72 (57, 84)<br>33/46	93 (82, 99)<br>43/46	92 (78, 98)<br>33/36	77 (64, 87)<br>43/56	67 (52, 80)<br>33/49<br>p=0.642[*]	93 (81, 99)<br>40/43<br>p=0.068[*]	92 (78, 98)<br>33/36<br>p>0.999[*]	71 (58, 83)<br>40/56<br>p=0.518[*]	64 (50, 76)<br>35/55<br>p=0.691[±]<br>p=0.387[¥]	97 (86, 100)<br>36/37<br>p=0.382[±]<br>p=0.419[¥]	97 (85, 100)<br>35/36<br>p=0.304[±]<br>p=0.304[¥]	64 (50, 77)<br>36/56<br>p=0.418[±]<br>p=0.147[¥]
**Ultra**	91 (79, 98)<br>42/46<br>**p=0.016**[‡]	76 (61, 87)<br>35/46<br>**p=0.020**[‡]	79 (66, 89)<br>42/53<br>p=0.114[‡]	90 (76, 97)<br>35/39<br>p=0.105[‡]	88 (75, 95)<br>43/49<br>**p=0.016**[‡]<br>p=0.573[*]	77 (61, 88)<br>33/43<br>**p=0.035**[‡]<br>p=0.942[*]	81 (68, 91)<br>43/53<br>p=0.167[‡]<br>p=0.808[*]	85 (69, 94)<br>33/39<br>p=0.134[‡]<br>p=0.498[*]	84 (71, 92)<br>46/55<br>**p=0.017**[‡]<br>p=0.551[±]<br>p=0.252[¥]	81 (65, 92)<br>30/37<br>**p=0.025**[‡]<br>p=0.636[±]<br>p=0.583[¥]	87 (75, 95)<br>46/53<br>p=0.091[‡]<br>p=0.427[±]<br>p=0.301[¥]	77 (61, 89)<br>30/39<br>p=0.188[‡]<br>p=0.389[±]<br>p=0.129[¥]

Within rows: [*]MRS vs. eMRS, eMRS vs. CRS[±], MRS vs. CRS[¥]; Within columns: Xpert vs. Ultra[‡]

Abbreviations: CI, confidence interval; CRS, composite reference standard; eMRS, extended microbiological reference standard; MRS, microbiological reference standard; NPV, Negative predictive value; PPV, Positive predictable value; Ultra, Xpert MTB/RIF Ultra; Xpert, Xpert MTB/RIF.

Supplementary Table 3: Per patient information for study Ultra-positive patients that were MRS -negative (culture- and cytology-negative) with information on their Ultra semi-quantitation category, previous TB status, TB treatment initiation status and patient's status after at least 12-weeks of follow-up. Data are n/N (%).

**Patient ID**	**Previous TB**	**Study Ultra semi-quantitation category**	**Routine PCR result**	**Treatment initiated after 12-week follow up**	**Did the patient get better?** (asked telephonically if patient started treatment)
FNAB038	No	Trace	Xpert-negative	No	N/A
FNAB060	No	Very Low	Xpert-negative	Yes	Yes
FNAB072	No	Very Low	Xpert-negative	No	N/A
FNAB076	No	Medium	Xpert-positive (Low)	Yes	Yes
FNAB110	No	Trace	Xpert-negative	No	N/A
FNAB114	No	Medium	Xpert-positive (Medium)	No	N/A
FNAB128	No	Trace	Xpert-negative	No	N/A
FNAB132	No	Trace	Xpert-negative	No	N/A
FNAB172	No	Low	Xpert-negative	Yes	Yes
FNAB180	Yes	Low	Xpert-negative	No	N/A
FNAB200	No	Low	Xpert-positive (Low)	Yes	Yes
FNAB206	Yes	Trace	Not done	No	N/A
FNAB210	Yes	Trace	Ultra-negative	No	N/A
FNAB214	No	Trace	Ultra-negative	No	N/A
FNAB218	Yes	Trace	Ultra-negative	No	N/A
FNAB220	No	Trace	Ultra-positive (Very low)	Yes	Yes
FNAB230	No	Trace	Ultra-negative	No	N/A
FNAB232	Yes	Medium	Ultra-positive (Medium)	Yes	Yes
FNAB245	No	Trace	Ultra-negative	No	N/A
FNAB251	Yes	Trace	Ultra-negative	No	N/A
FNAB273	No	Trace	Ultra-negative	No	N/A
FNAB403	No	Medium	Ultra-positive (Medium)	No	N/A
**Overall**	6/22 (27)	Trace: 13/22 (59)<br>Very low: 2/22 (9)<br>Low: 3/22 (14)<br>Medium: 4/22 (18)	Xpert: 11/21 (52) positive, 3/11 (27) negative, 8/11 (73)<br>Ultra: 10/21 (48) positive, 3/10 (30) negative, 7/10 (70)	6/22 (27)	6/6 (100)

Missing data: Routine PCR not done, n=1.

Abbreviations: FNAB, fine needle aspirate biopsy; PCR, polymerase chain reaction; Ultra, Xpert MTB/RIF Ultra.

If the patient was not initiated on TB treatment, N/A was recorded in the last column.

Supplementary Table 4: Diagnostic accuracy of Ultra on urine or FNABs measured using the MRS in a head-to-head analysis stratified by HIV status. Urine Ultra had lower sensitivity than FNAB Ultra but increased specificity **(Table 2)**. Data are %, 95% CI, and n/N

	**All patients**				**HIV-negative**				**HIV-positive**			
	**n=76**				**n=18/75 (24)**				**n=57/75 (76)**			
	**Sensitivity**	**Specificity**	**PPV**	**NPV**	**Sensitivity**	**Specificity**	**PPV**	**NPV**	**Sensitivity**	**Specificity**	**PPV**	**NPV**
**Urine-Ultra**	18 (7, 35)<br>6/33	98 (88, 100)<br>42/43	86 (42, 100)<br>6/7	61 (48, 72)<br>42/69	0 (0, 52)<br>0/5	100 (75, 100)<br>13/13	0/0	72 (47, 90)<br>13/18	21 (8, 41)<br>6/28<br>p=0.252*	97 (82, 100)<br>28/29<br>p=498*	86 (42, 100)<br>6/7	56 (41, 70)<br>28/50<br>p=0.228*
**Study FNAB-Ultra**	91 (76, 98)<br>30/33<br>**p<0.001**‡	60 (44, 75)<br>26/43<br>**p<0.001**‡	64 (49, 77)<br>30/47<br>p=0.252‡	90 (73, 98)<br>26/29<br>**p=0.005**‡	80 (28, 99)<br>4/5<br>**p=0.010**‡	38 (14, 68)<br>5/13<br>**p=0.001**‡	33 (10, 65)<br>4/12	83 (36, 100)<br>5/6<br>p=0.586‡	93 (76, 99)<br>26/28<br>**p<0.001**‡<br>p=0.357*	69 (49, 85)<br>20/29<br>**p=0.005**‡<br>p=0.063*	74 (57, 88)<br>26/35<br>p=0.517‡<br>**p=0.011***	91 (71, 99)<br>20/22<br>**p=0.004**‡<br>p=0.595*

Missing data: Non-actionable Ultras (n=1), no HIV (n=1) in the head-to-head table.
Within column p-values: ‡ Urine-Ultra vs. FNAB-Ultra
Within row p-values: *HIV-negative vs. HIV-positive
Abbreviations: CI, confidence interval; CRS, composite reference standard; eMRS, extended microbiological reference standard; MRS, microbiological reference standard; NPV, negative predictive value; PPV, positive predictive value; Xpert, Xpert MTB/RIF; Ultra, Xpert MTB/RIF Ultra

Supplementary Table 5: Non-head-to-head and head-to-head diagnostic accuracy analyses of Xpert and Ultra using a MRS, eMRS and CRS for the detection of *Mycobacterium tuberculosis complex* DNA with and without exclusion of Ultra trace results. Routine Xpert results were compared to study Ultra results. Ultra has similar diagnostic accuracy compared to Xpert after trace positive exclusion. Study Ultra results with trace excluded were like study Ultra results. Similar trends are seen across reference standards. Data are %, 95% CI, and n/N.

	**Non-head-to-head**											
	**MRS**				**eMRS**				**CRS**			
	**n=96**				**n=97**				**n=97**			
	**Sensitivity**	**Specificity**	**PPV**	**NPV**	**Sensitivity**	**Specificity**	**PPV**	**NPV**	**Sensitivity**	**Specificity**	**PPV**	**NPV**
**Xpert[ω]**	73 (58, 85)<br>35/48	92 (80, 98)<br>44/48	90 (76, 97)<br>35/39	77 (64, 87)<br>44/57	69 (55, 81)<br>36/52<br>p=0.685[*]	91 (79, 98)<br>41/45<br>p=0.924[*]	90 (76, 97)<br>36/40<br>p=0.510[*]	72 (58, 83)<br>41/57<br>p=0.519[*]	65 (52, 77)<br>39/60<br>p=0.635[±]<br>p=0.379[¥]	97 (86, 100)<br>36/37<br>p=0.244[±]<br>p=0.274[¥]	98 (87, 100)<br>39/40<br>p=0.166[±]<br>p=0.157[¥]	63 (49, 76)<br>36/57<br>p=0.317[±]<br>p=0.102[¥]
	**n=111**				**n=112**				**n=112**			
**Ultra excluding trace**	83 (71, 92)<br>45/54<br>p=0.201[‡]	84 (72, 93)<br>48/57<br>p=0.248[‡]	83 (71, 92)<br>45/54<br>p=0.379[‡]	84 (72, 93)<br>48/57<br>p=0.343[‡]	81 (69, 90)<br>47/58<br>p=0.151[‡]<br>p=0.751[*]	85 (73, 93)<br>46/54<br>p=0.368[‡]<br>p=0.887[*]	85 (73, 94)<br>47/55<br>p=0.510[‡]<br>p=0.760[*]	81 (68, 90)<br>46/57<br>p=0.271[‡]<br>p=0.622[*]	74 (62, 84)<br>51/69<br>p=0.272[‡]<br>p=0.341[±]<br>p=0.210[¥]	91 (78, 97)<br>39/43<br>p=0.224[‡]<br>p=0.413[±]<br>p=0.340[¥]	93 (82, 98)<br>51/55<br>p=0.304[‡]<br>p=0.221[±]<br>p=0.130[¥]	68 (55, 80)<br>39/57<br>p=0.554[‡]<br>p=0.132[±]<br>**p=0.047[¥]**
**Δ Trace excluded[Φ]**	-2 (-15, 12)<br>p=0.808[§]	+15 (1, 30)<br>**p=0.041[§]**	+13 (-1, 28)<br>p=0.081[§]	0 (-13, 13)<br>p>0.999[§]	-2 (-15, 12)<br>p=0.799[§]	+16 (2, 31)<br>**p=0.034[§]**	+13 (-0.03, 28)<br>p=0.063[§]	0 (-14, 14)<br>p>0.999[§]	-2 (-16, 12)<br>p=0.738[§]	+20 (5, 35)<br>**p=0.016[§]**	+15 (3, 26)<br>**p=0.026[§]**	0 (-17, 17)<br>p>0.999[§]
	**Head-to-head**											
	**n=82**				**n=82**				**n=82**			
**Xpert[ω]**	80 (64, 91)<br>32/40	93 (81, 99)<br>39/42	91 (77, 98)<br>32/35	83 (69,92)<br>39/47	74 (59, 86)<br>32/43<br>p=0.545[*]	92 (79, 98)<br>36/39<br>p=0.925[*]	91 (77, 98)<br>32/35<br>p>0.999[*]	77 (62, 88)<br>36/47<br>p=0.441[*]	69 (55, 82)<br>34/49<br>p=0.593[±]<br>p=0.255[¥]	97 (84, 100)<br>32/33<br>p=0.390[±]<br>p=0.431[¥]	97 (85, 100)<br>34/35<br>p=0.303[±]<br>p=0.303[¥]	68 (53, 81)<br>32/47<br>p=0.356[±]<br>p=0.093[¥]
**Ultra excluding trace**	90 (76, 97)<br>36/40<br>p=0.210[‡]	83 (69, 93)<br>35/42<br>p=0.178[‡]	84 (69, 93)<br>36/43<br>p=0.311[‡]	90 (76, 97)<br>35/39<br>p=0.367[‡]	86 (72, 95)<br>37/43<br>p=0.176[‡]<br>p=0.580[*]	85 (69, 94)<br>33/39<br>p=0.288[‡]<br>p=0.875[*]	86 (72, 95)<br>37/43<br>p=0.459[‡]<br>p=0.763[*]	85 (69, 94)<br>33/39<br>p=0.353[‡]<br>p=0.498[*]	82 (68, 91)<br>40/49<br>p=0.159[‡]<br>p=0.567[±]<br>p=0.266[¥]	91 (76, 98)<br>30/33<br>p=0.302[‡]<br>p=0.421[±]<br>p=0.338[¥]	93 (81, 99)<br>40/43<br>p=0.412[‡]<br>p=0.291<br>p=0.178[¥]	77 (61, 89)<br>30/39<br>p=0.363[‡]<br>p=0.389[±]<br>p=0.129[¥]
**Δ Trace excluded[Φ]**	-1 (-14, 11)<br>p=0.836[§]	+7 (-9, 24)<br>p=0.400[§]	+5 (-11, 20)<br>p=0.576[§]	0 (-13, 13)<br>p>0.999[§]	-2 (-16, 12)<br>p=0.808[§]	+8 (-9, 25)<br>p=0.369[§]	+5 (-10, 20)<br>p=0.521[§]	0 (-16, 16)<br>p=0.484[§]	-2 (-17, 13)<br>p=0.788[§]	+10 (-6, 26)<br>p=0.241[§]	+6 (-6, 18)<br>p=0.320[§]	0 (-18, 18)<br>p>0.999[§]

Within column p-values: [‡]Xpert vs. Ultra within an analysis (non-head-to-head or head-to-head) in patients of the same HIV status (overall, negative, or positive), [§]Study Ultra results (Supplementary Table 2) vs. study Ultra results excluding trace results within an analysis (non-head-to-head or head-to-head) in patients using different reference standards (MRS, eMRS, or CRS).

Within row p-values: [*]MRS vs. eMRS, [±]eMRS vs. CRS, [¥]MRS vs. CRS within an analysis (non-head-to-head or head-to-head).

[ϖ]Although Xpert data are already shown in Supplementary Table 2, small differences in the number of samples included occur in the head-to-head comparison. For the non-head-to-head comparison the Xpert data are identical to that in Supplementary Table 2 but are included here for readability.

[ɸ]This comparison is Ultra with traces excluded vs. Ultra with traces included and considered positive.

Abbreviations: CRS, composite reference standard; eMRS, extended microbiological reference standard; MRS, microbiological reference standard; Ultra, Xpert MTB/RIF Ultra; Xpert, Xpert MTB/RIF.

Supplementary Table 6: Non-head-to-head and head-to-head diagnostic accuracy analyses of Xpert and Ultra using a MRS, eMRS and CRS for the detection of Mycobacterium tuberculosis complex DNA, reclassifying trace positive Ultra results as negative. Routine Xpert results were compared to study Ultra results. Ultra has similar diagnostic accuracy compared to Xpert when trace positive results are reclassified as negative. Study Ultra results with trace reclassified had increased sensitivity and decreased specificity compared to normal study Ultra results. Similar trends are seen across reference standards. Data are %, 95% CI, and n/N.

	**Non-head-to-head**											
	**MRS**				**eMRS**				**CRS**			
	**n=96**				**n=97**				**n=97**			
	**Sensitivity**	**Specificity**	**PPV**	**NPV**	**Sensitivity**	**Specificity**	**PPV**	**NPV**	**Sensitivity**	**Specificity**	**PPV**	**NPV**
**Xpert[ω]**	73 (58, 85)<br>35/48	92 (80, 98)<br>44/48	90 (76, 97)<br>35/39	77 (64, 87)<br>44/57	69 (55, 81)<br>36/52<br>p=0.685[*]	91 (79, 98)<br>41/45<br>p=0.924[*]	90 (76, 97)<br>36/40<br>p=0.510[*]	72 (58, 83)<br>41/57<br>p=0.519[*]	65 (52, 77)<br>39/60<br>p=0.635[±]<br>p=0.379[¥]	97 (86, 100)<br>36/37<br>p=0.244[±]<br>p=0.274[¥]	98 (87, 100)<br>39/40<br>p=0.166[±]<br>p=0.157[¥]	63 (49, 76)<br>36/57<br>p=0.317[±]<br>p=0.102[¥]
	**n=130**				**n=131**				**n=131**			
**Ultra with trace reclassified**	75 (62, 85)<br>45/60<br>p=0.806[‡]	87 (77, 94)<br>61/70<br>p=0.441[‡]	83 (71, 92)<br>45/54<br>p=0.379[‡]	80 (70, 89)<br>61/76<br>p=0.667[‡]	73 (61, 84)<br>47/64<br>p=0.618[‡]<br>p=0.843[*]	88 (78, 95)<br>59/67<br>p=0.609[‡]<br>p=0.871[*]	85 (73, 94)<br>47/55<br>p=0.510[‡]<br>p=0.760[*]	78 (67, 86)<br>59/76<br>p=0.451[‡]<br>p=0.691[*]	67 (55, 77)<br>51/76<br>p=0.797[‡]<br>p=0.415[±]<br>p=0.316[¥]	93 (82, 98)<br>51/55<br>p=0.343[‡]<br>p=0.389[±]<br>p=0.310[¥]	93 (82, 98)<br>51/55<br>p=0.304[‡]<br>p=0.221[±]<br>p=0.130[¥]	67 (55, 77)<br>51/76<br>p=0.636[‡]<br>p=0.147[±]<br>p=0.066[¥]
**Δ Trace reclassified[ϕ]**	-10 (-19, -1)<br>**p=0.014[§]**	+18 (8, 29)<br>**p<0.001[§]**	+13 (-1, 28)<br>p=0.081[§]	-4 (-17, 9)<br>p=0.558[§]	+10 (-18, 1)<br>**p=0.014[§]**	+19 (8, 30)<br>**p<0.001[§]**	+13 (-0.03, 28) p=0.063[§]	-3 (-17, 11)<br>p=0.667[§]	-9 (-17, 1)<br>**p=0.008[§]**	+22 (9, 35)<br>**p=0.001[§]**	+15 (3, 26)<br>**p=0.026[§]**	+1 (-17, 14)<br>p=0.873[§]
	**Head-to-head**											
	**n=92**				**n=92**				**n=92**			
**Xpert[ω]**	72 (57, 84)<br>33/46	93 (82, 99)<br>43/46	92 (78, 98)<br>33/36	77 (64, 87)<br>43/56	67 (52, 80)<br>33/49<br>p=0.642[*]	93 (81, 99)<br>40/43<br>p=0.932[*]	92 (78, 98)<br>33/36<br>p>0.999[*]	71 (58, 83)<br>40/56<br>p=0.518[*]	64 (50, 76)<br>35/55<br>p=0.691[±]<br>p=0.387[¥]	97 (86, 100)<br>36/37<br>p=0.382[±]<br>p=0.419[¥]	97 (85, 100)<br>35/36<br>p=0.304[±]<br>p=0.303[¥]	64 (50, 77)<br>36/56<br>p=0.418[±]<br>p=0.147[¥]
**Ultra with trace reclassified**	78 (64, 89)<br>36/46<br>p=0.470[‡]	85 (71, 94)<br>39/46<br>p=0.180[‡]	84 (69, 93)<br>36/43<br>p=0.290[‡]	80 (66, 90)<br>39/49<br>p=0.729[‡]	76 (61, 87)<br>37/49<br>p=0.371[‡]<br>p=0.751[*]	86 (72, 95)<br>37/43<br>p=0.291[‡]<br>p=0.763[*]	86 (72, 95)<br>37/43<br>p=0.434[‡]<br>p=0.763[*]	76 (61, 87)<br>37/49<br>p=0.637[‡]<br>p=0.498[*]	73 (59, 84)<br>40/55<br>p=0.306[‡]<br>p=0.747[±]<br>p=0.521[¥]	92 (78, 98)<br>34/37<br>p=0.304[‡]<br>p=0.409[±]<br>p=0.323[¥]	93 (81, 99)<br>40/43<br>p=0.397[‡]<br>p=0.291[±]<br>p=0.178[¥]	69 (55, 82)<br>34/49<br>p=0.580[‡]<br>p=0.489[±]<br>p=0.247[¥]
**Δ Trace reclassified[ϕ]**	-13 (-25, 1)<br>**p=0.014[§]**	+9 (-2, 19)<br>**p=0.046[§]**	+5 (-11, 20)<br>p=0.576[§]	-10 (-25, 5)<br>p=0.196[§]	-12 (-23, -1)<br>**p=0.014[§]**	+9 (-2, 20)<br>**p=0.046[§]**	+5 (-10, 20)<br>p=0.521[§]	-9 (-26, 7)<br>p=0.293[§]	-11 (-21, -1)<br>**p=0.014[§]**	+11 (-2, 24)<br>**p=0.046[§]**	+6 (-6, 18)<br>p=0.320[§]	-8 (-26, 11)<br>p=0.430[§]

Within column p-values: [‡]Xpert vs. Ultra within an analysis (non-head-to-head or head-to-head) in patients of the same HIV status (overall, negative, or positive), [§]Study Ultra results (Supplementary Table 2) vs. study Ultra results excluding trace results within an analysis (non-head-to-head or head-to-head) in patients using different reference standards (MRS, eMRS, or CRS).

Within row p-values: [*]MRS vs. eMRS, [±]eMRS vs. CRS, [¥]MRS vs. CRS within an analysis (non-head-to-head or head-to-head).

[ϖ]Although Xpert data are already shown in Supplementary Table 2, small differences in the number of samples included occur in the head-to-head comparison. For the non-head-to-head comparison the Xpert data are identical to that in Supplementary Table 2 but are included here for readability.

[ϕ]This comparison is Ultra with traces reclassified as negative vs. Ultra with traces considered positive.

Abbreviations: CRS, composite reference standard; eMRS, extended microbiological reference standard; MRS, microbiological reference standard; Ultra, Xpert MTB/RIF Ultra; Xpert, Xpert MTB/RIF.
